# Qualitative insights into post-dieting strategies: reverse dieting, maintenance, and ad libitum approaches following caloric restriction: a preliminary analysis

**DOI:** 10.1080/15502783.2025.2550189

**Published:** 2025-08-26

**Authors:** Sydney Monahan, Valentina Rodriguez Da Silva, Sofia Atehortua, Avinash Konapala, Eric T. Trexler, Landon Shannahan, Wayne A. Ayers-Creech, Cassidy Bale, Mateus Muniz, Grace Chong, Bill I. Campbell

**Affiliations:** a University of South Florida, Exercise Science & Kinesiology Program, Performance & Physique Enhancement Lab, Tampa, FL, USA; b Duke University, Department of Evolutionary Anthropology, Durham, NC, USA

**Keywords:** Caloric intake, hunger, fullness, post-diet strategy

## Abstract

**Background:**

While post-dieting strategies focus on minimizing fat regain, they often neglect the individual’s subjective experience. This study explored how three different post-dieting approaches impact subjective outcomes.

**Methods:**

This was a randomized, parallel-group study consisting of resistance-trained males and females. Upon reaching 5% bodyweight loss, participants began one of three post-diet strategies: a gradual weekly increase in calories of 8.5% for males and 11.7% for females (REV: *n* = 35, 5 males, 30 females), immediate return to estimated maintenance calories based on the Hall equation (NIH, *n* = 31), or ad libitum control (CON, *n* = 24), all of which were followed for 15 weeks. A 12-item, 7-point Likert-scale survey assessing subjective psychological and physical responses was distributed weekly via Qualtrics (Qualtrics, Provo, UT, USA). Data was analyzed via repeated measures ANOVA.

**Results:**

Of the twelve questions administered, only four had responses that significantly differed between groups and across time. CON reported a significantly greater ease of sticking to the diet than REV from weeks 10–15 (*p* ≤ 0.05). NIH reported a significantly greater ease of sticking to the diet than REV from weeks 14–15 (*p* < 0.05). NIH reported lower hunger than REV during weeks 1–2 (*p* ≤ 0.05). Both CON and NIH reported significantly higher hunger than REV during weeks 6–15 and during weeks 8–15, respectively (*p* ≤ 0.05). NIH reported greater fullness than both REV and CON during weeks 1–2 (*p* ≤ 0.05) and during weeks 1–6, respectively (*p* ≤ 0.05). REV reported greater fullness than the NIH and CON in the later stages of the study, weeks 8–15 (*p* ≤ 0.05) and during weeks 5–15 (*p* ≤ 0.05), respectively. CON reported a stronger desire to eat than REV during weeks 6–15 (*p* ≤ 0.05), while NIH reported a stronger desire to eat than REV during weeks 8–15 (*p* ≤ 0.05). No other significant differences emerged between groups for any other time points or questions.

**Conclusion:**

Post-dieting strategies significantly influenced subjective experiences of ease, hunger, fullness, and desire to eat in the post-diet period. This became especially evident in the latter half of the study when post-dieting experiences mirrored the relative caloric prescription. REV initially showed higher hunger levels, lower fullness, and stronger desire to eat, however, by weeks 5–8, as caloric intake continued to increase, REV participants reported greater fullness, lower hunger, and a lower desire to eat. This suggests that while subjective responses are consistent with caloric intake, no single post-dieting strategy is universally superior. Instead, strategy selection should be guided by individual client preferences to support long-term success.

